# 加速溶剂提取-同位素稀释-高分辨气相色谱-高分辨质谱法测定生物样品中82种多氯联苯

**DOI:** 10.3724/SP.J.1123.2021.10018

**Published:** 2022-05-08

**Authors:** Yinju WU, Bailu QU, Yulan HOU, Haibin YU, Renji XU, Xiaoyan ZHENG

**Affiliations:** 1.中国环境监测总站, 北京 100012; 1. China National Environmental Monitoring Centre, Beijing 100012, China; 2.湖南省长沙生态环境监测中心, 湖南 长沙 410000; 2. Hunan Changsha Eco-Environment Monitoring Center, Changsha 410000, China

**Keywords:** 加速溶剂提取, 同位素稀释-高分辨气相色谱-高分辨质谱, 环境监测, 多氯联苯, 鱼, 贝, accelerated solvent extraction (ASE), isotope dilution-high resolution gas chromatography-high resolution mass spectrometry (ID-HRGC-HRMS), environmental monitoring, polychlorinated biphenyls (PCBs), fish, shellfish

## Abstract

我国水产品中多氯联苯(PCBs)的检测方法,主要以6种指示性PCBs和12种二噁英类共平面PCBs为主,仅涵盖有限的PCBs。为更全面地获得生物体中PCBs的浓度水平,深入探讨PCBs在生物体内的代谢和富集特征,进而准确评价PCBs对人类的暴露水平及风险,以鱼和贝类作为生物样品代表,建立了加速溶剂提取-同位素稀释-高分辨气相色谱-高分辨质谱(ASE-ID-HRGC-HRMS)测定生物样品中82种PCBs的方法。比较了振荡提取和加速溶剂提取两种提取方式的回收率和重复性,最终采用正己烷-二氯甲烷(1∶1, v/v)对PCBs进行加速溶剂提取。考察了各流分淋洗液对PCBs的回收率,确定了样品提取液经8 g 44%酸性硅胶层析柱(内径15 mm), 90 mL正己烷洗脱的净化方式。样品提取液净化浓缩后进行HRGC-HRMS分析,色谱柱采用DB-5MS超低流失石英毛细管柱(60 m×0.25 mm×0.25 μm)。通过优化后的升温程序对化合物进行分离,以保留时间和两个特征离子精准定性,采用同位素内标法定量。结果表明,在0.1~200 μg/L范围内,平均相对响应因子(RRF)的相对标准偏差值(RSD, *n*=7)均≤20%,相关系数(*r*^2^)>0.99。生物样品中PCBs的方法检出限为0.02~3 pg/g;鱼类中PCBs平均加标回收率为71.3%~141%, RSD(*n*=7)为2.1%~14%;贝类中PCBs平均加标回收率为76.9%~143%, RSD为1.4%~11%。该方法灵敏、准确、可靠,可以更加全面具体地分析鱼和贝类等水产品受PCBs的污染情况,为国内外开展生物监测提供有效的技术支持,从而服务于相关生态环境管理及履行《斯德哥尔摩公约》。

## Abstract (en)

In China, the detection methods for polychlorinated biphenyls (PCBs) in aquatic products are mainly effective for 6 indicative PCBs and 12 coplanar dioxin-like PCBs, which only account for a limited proportion of PCBs in organisms. In this study, to obtain the detailed concentration levels of PCBs in organisms, elucidate the metabolism and enrichment characteristics of PCBs in organisms, and accurately evaluate the exposure level and risks of PCBs to humans, an improved method for the simultaneous determination of 82 PCBs in fish and shellfish samples was developed using isotope dilution-high resolution gas chromatography-high resolution mass spectrometry (ID-HRGC-HRMS). The recovery and reproducibility of two extraction methods, i. e., oscillatory extraction and accelerated solvent extraction (ASE), were compared. Finally, ASE was chosen for subsequent experiments. Specifically, after adding 1 ng^13^C-labeled extraction internal standards, the samples were extracted under pressure by ASE using a mixture of *n*-hexane-dichloromethane (1∶1, v/v). The experimental conditions employed for this were a pressure of 10.3 MPa, heating temperature of 100 ℃, heating time of 5 min, static time of 8 min, flush volume of 60%, purging time of 120 s, and 34 mL cells. Subsequently, the extracts were loaded on an 8 g acid silica gel (44%) column (inner diameter: 15 mm) and eluted with 90 mL of *n*-hexane. After purification and concentration, the analytes were determined by HRGC-HRMS with a fused-silica capillary column (DB-5MS, 60 m×0.25 mm×0.25 μm). The temperature program was optimized to separate the most target compounds at the baseline. Specifically, the initial oven temperature was 120 ℃, which was held for 1 min, following by heating to 180 ℃ at 30 ℃/min, heating to 210 ℃ at 2 ℃/min and holding for 1 min, and further heating to 310 ℃ at 2.5 ℃/min and holding for 1 min. The injector and ion source temperatures were 270 ℃ and 280 ℃, respectively. With a static resolution of 10000, the HRMS instrument was operated in the selected-ion monitoring mode at an electron energy of 35 eV. The 82 PCBs were qualified by their retention time and two characteristic ions, and thereafter quantified using the mean relative corresponding factor (RRF). The results showed that the relative standard deviation (RSD) of the RRF obtained from six-point calibration standard solutions was less than 20%. The linearity ranges were from 0.1 to 200 μg/L, and the correlation coefficients (*r*^2^) were greater than 0.99. Under optimum conditions, the method detection limits (MDLs) for the PCBs of biological samples were in the range of 0.02-3 pg/g. To validate the method, the fish and shellfish samples were spiked with a low level (0.4 ng) and high level (3.6 ng) of native PCB standards. The spiked recoveries using low-concentration native PCBs were 71.3%-139% in fish and 76.9%-143% in shellfish, and the RSDs (*n*=7) were 2.1%-14% and 4.5%-14%, respectively. The spiked recoveries using high-concentration native PCBs were 77.6%-141% and 82.2%-131%, respectively, and the RSDs (*n*=7) were 1.4%-9.4% and 1.7%-11%, respectively. An analysis of fresh fish and shellfish samples showed that the contents of a single PCB ranged from “not detected” to 54.1 pg/g, where 12 coplanar dioxin-like PCBs were detected in the range of 12.6 pg/g to 74.5 pg/g, six indicative PCBs in the range of 30.9 pg/g to 62.1 pg/g, and 82 PCBs in the range of 174 pg/g to 672 pg/g. It was concluded that this method could be successfully applied for the determination of PCBs in biological samples with good accuracy and precision. This comprehensive analytical method of PCBs in aquatic products provides effective technical support for biological monitoring; it will also aid in ecological and environmental management and the implementation of the Stockholm Convention policies.

多氯联苯(polychlorinated biphenyls, PCBs)是联苯苯环上氢原子被氯原子取代的化合物的总称,其分子式为C_12_H_(10-_*_n_*_)_Cl*_n_*。根据氯原子取代数和取代位置的不同,PCBs共有209种同类物,国际理论和应用化学联合会(International Union of Pure and Applied Chemistry, IUPAC)已对PCBs及其衍生物进行了系统的命名和编号。通常情况下,PCBs是一种无色或浅黄色的油状物质,有非常稳定的物理化学性质,属半挥发或不挥发物质,蒸气压低,难溶于水,其作为优质工业添加剂,在石油产品、塑料、农药加工等行业大量使用。但PCBs在环境中难降解,残留时间长,易溶于脂肪,可在食物链中积累,且具有致畸性、致癌性、致突变性,是首批列入《关于持久性有机污染物的斯德哥尔摩公约》的12种优先控制污染物之一^[1]^。

膳食摄入是普通人群暴露PCBs的主要途径,占人体总暴露量的90%以上^[2]^。在各类膳食中,鱼类、贝类及其他水产品含有较高的PCBs^[3,4]^。我国水产品资源丰富,鱼、虾、贝类等水产品是餐桌常见食品,其受污染程度直接影响人体健康。同时,生物体内PCBs含量的高低往往能反映其所处环境的污染水平。实际销售的工业品中仅测定出130余种PCBs同类物^[2]^。全球环境监测系统/食品规划部分(GEMS/FOOD)认为,将食品中PCBs浓度用相应的工业品PCBs进行量化,可能会导致严重的定性和定量错误;分析特定的PCBs同类物能提供与暴露评估相关的更有意义的信息^[5]^。

目前,GEMS/FOOD要求分析的PCBs同类物包括12种二噁英类PCBs(PCB77、PCB81、PCB105、PCB114、PCB118、PCB123、PCB126、PCB156、PCB157、PCB167、PCB169和PCB189)、6种“指示性PCBs”(PCB28、PCB52、PCB101、PCB138、PCB153和PCB180)以及另外41种非二噁英类PCBs^[3]^。有关PCBs在生物体中的测定国内外均有报道^[6⇓⇓⇓⇓-11]^,主要以指示性PCBs和二噁英类PCBs为主。目前,国内颁布的动物源性食品中PCBs的标准分析方法,主要关注的是指示性PCBs、1种二噁英类PCB和另外16种非二噁英类PCBs,分析方法为气相色谱-电子捕获检测(GC-ECD)和气相色谱-质谱法(GC-MS),定量方法为内标法和同位素稀释法(ID)^[12]^。ECD虽然对PCBs有很高的灵敏度,但其是通过化合物的电负性产生的响应及标准物质的保留时间进行定性,在同样的保留时间有可能有其他物质共流出,在定性方面存在明显不足。GC-MS和GC-MS/MS虽然通过化合物的特征离子碎片增加了定性的准确性,但要准确区分具有相同整数质荷比离子的干扰物仍存在难度,而且灵敏度较低。而HRGC-HRMS可对选择离子的质量数精确到小数点后4位,可准确区分具有相同整数质荷比离子的干扰物,提高了定性定量的准确性及抗干扰能力。同位素稀释法被认为最高计量标准的化学测量方法,对痕量物质的检测灵敏度高,选择性强,准确度好。同位素稀释法和HRGC-HRMS结合对生物样品中痕量PCBs进行测定,在定量和定性方面有其独有的优势。

结合环境监测实际情况,本文以鱼和贝类为水产品代表,通过探讨研究样品提取方式、提取溶剂、样品净化及仪器条件等一些关键技术问题,建立了加速溶剂提取仪(ASE)提取样品,酸性硅胶柱净化,同位素稀释-高分辨气相色谱-高分辨质谱测定生物中82种PCBs的方法。

## 1 实验部分

### 1.1 仪器、试剂和材料

气相色谱仪(Agilent 7890A, Agilent公司,美国);高分辨质谱仪(AutoSpec Premier, Waters公司,美国),配有Masslynx 4.1软件,可根据谱图直接计算每个样品的方法检出限;加速溶剂提取仪(ASE350, Thermo公司,美国);旋转蒸发仪(N-1200B, Eyela公司,日本);氮吹仪(N-EVAP^TM^ 112, Organomation Associates公司,美国);高速组织匀浆机(上海标本模型厂,DS-1);实验所用玻璃器皿使用前均用丙酮和正己烷各洗3次,使用后用有机溶剂涮洗,再用碱性洗涤液超声清洗或洗瓶机清洗,空白检测无残留。

二氯甲烷、正己烷和丙酮均为农残级(J. T. Baker公司,美国);壬烷(色谱纯,百灵威公司,德国);超纯水为二次去离子水;浓硫酸(优级纯,北京化工厂);无水硫酸钠(优级纯,天津市津科精细化工研究所), 450 ℃下烘4 h;硅胶(层析柱用,Merck公司,德国),粒径为0.063~0.100 mm, 550 ℃下烘12 h, 180 ℃下平衡1 h,冷却后装入密封的玻璃瓶中,并存放于干燥器中。44%酸性硅胶,为44 g浓硫酸与56 g活化硅胶充分混匀^[13]^。

PCBs标准系列(Wellington公司,加拿大):包括PCBs校准溶液系列(PCB-CVS-H,天然化合物质量浓度依次为0.1、0.5、2.0、10.0、40.0和200 ng/mL,相应的^13^C同位素提取内标和进样内标浓度均为50 ng/mL;壬烷),以及配套的82种天然化合物(PCB-PAR-H, 500 ng/mL;壬烷)、^13^C同位素提取内标(PCB-LCS-H, 1000 ng/mL;壬烷)和^13^C同位素进样内标(PCB-ISS-H, 1000 ng/mL;壬烷)均为直接购买的标准溶液。具体化合物信息见附表1(详见 https://www.chrom-China.com)。

**表1:** 多氯联苯的扫描窗口、特征离子、理论丰度比、保留时间、保留时间参考和定量参考

Scan window/ min		Compound	Qualitative ion		Quantitative ions		Theoretical ratio	*t*_R_/min	Retention time/ quantitation references
10.00-12.80		^13^C_12_-PCB1^*^	-		200.0795^a^, 202.0766^b^		3.13	10.76	^13^C_12_-PCB9/9
		PCB1	-		188.0393^a^, 190.0363^b^		3.13	10.76	^13^C_12_-PCB1/9
		^13^C_12_-PCB3^*^	-		200.0795^a^, 202.0766^b^		3.13	12.41	^13^C_12_-PCB9/9
		PCB3	-		188.0393^a^, 190.0363^b^		3.13	12.42	^13^C_12_-PCB3/3
		PFK	218.9856		-		-	-	-
12.80-19.10		^13^C_12_-PCB4^*^	-		234.0406^a^, 236.0376^b^		1.56	13.19	^13^C_12_-PCB9/9
		PCB4/10	225.9944^c^		222.0003^a^, 223.9974^b^		1.56	13.20	^13^C_12_-PCB4/4
		^13^C_12_-PCB9^**^	-		234.0406^a^, 236.0376^b^		1.56	14.38	-
		PCB6	225.9944^c^		222.0003^a^, 223.9974^b^		1.56	14.90	^13^C_12_-PCB4/4
		^13^C_12_-PCB8^*^	-		234.0406^a^, 236.0376^b^		1.56	15.24	^13^C_12_-PCB9/9
		PCB8	225.9944^c^		222.0003^a^, 223.9974^b^		1.56	15.26	^13^C_12_-PCB8/8
		^13^C_12_-PCB19^*^	-		268.0016^a^, 269.9986^b^		1.04	16.22	^13^C_12_-PCB9/37
		PCB19	-		255.9613^a^, 257.9584^b^		1.04	16.23	^13^C_12_-PCB19/19
		PCB18	-		255.9613^a^, 257.9584^b^		1.04	17.50	^13^C_12_-PCB19/19
		^13^C_12_-PCB15^*^	-		234.0406^a^, 236.0376^b^		1.56	17.87	^13^C_12_-PCB9/9
		PCB15	225.9944^c^		222.0003^a^, 223.9974^b^		1.56	17.90	^13^C_12_-PCB15/15
		PCB16	-		255.9613^a^, 257.9584^b^		1.04	18.62	^13^C_12_-PCB19/19
		PFK	242.9856		-		-	-	-
19.10-26.10		^13^C_12_-PCB54^*^	-		301.9626^a^, 303.9597^b^		0.77	19.48	^13^C_12_-PCB37/79
		PCB54	293.9165^c^		289.9224^a^, 291.9194^b^		0.77	19.51	^13^C_12_-PCB54/54
		^13^C_12_-PCB28^*^	-		268.0016^a^, 269.9986^b^		1.04	20.51	^13^C_12_-PCB37/37
		PCB28/31	259.9554^c^		255.9613^a^, 257.9584^b^		1.04	20.53	^13^C_12_-PCB28/28
		PCB33	259.9554^c^		255.9613^a^, 257.9584^b^		1.04	21.10	^13^C_12_-PCB28/28
		PCB22	259.9554^c^		255.9613^a^, 257.9584^b^		1.04	21.65	^13^C_12_-PCB28/28
		^13^C_12_-PCB52^*^	-		301.9626^a^, 303.9597^b^		0.77	22.77	^13^C_12_-PCB79/79
		PCB52	293.9165^c^		289.9224^a^, 291.9194^b^		0.77	22.79	^13^C_12_-PCB52/52
		PCB49	293.9165^c^		289.9224^a^, 291.9194^b^		0.77	23.07	^13^C_12_-PCB52/52
		^13^C_12_-PCB104^*^	-		337.9207^b^, 339.9178^c^		1.55	23.77	^13^C_12_-PCB37/111
		PCB104	323.8834^a^		325.8804^b^, 327.8775^c^		1.55	23.80	^13^C_12_-PCB104/104
		PCB44	293.9165^c^		289.9224^a^, 291.9194^b^		0.77	24.16	^13^C_12_-PCB52/52
		^13^C_12_-PCB37^**^	-		268.0016^a^, 269.9986^b^		1.04	24.63	-
		PCB37	259.9554^c^		255.9613^a^, 257.9584^b^		1.04	24.64	^13^C_12_-PCB37/28
		PCB41	293.9165^c^		289.9224^a^, 291.9194^b^		0.77	25.02	^13^C_12_-PCB52/52
		PCB40	293.9165^c^		289.9224^a^, 291.9194^b^		0.77	25.55	^13^C_12_-PCB52/52
		PFK	280.9825		-		-	-	-
26.10-33.85		PCB74	293.9165^c^		289.9224^a^, 291.9194^b^		0.77	26.79	^13^C_12_-PCB70/70
		^13^C_12_-PCB70^*^	-		301.9626^a^, 303.9597^b^		0.77	27.08	^13^C_12_-PCB79/79
		PCB70	293.9165^c^		289.9224^a^, 291.9194^b^		0.77	27.09	^13^C_12_-PCB70/70
		^13^C_12_-PCB95^*^	-		337.9207^b^, 339.9178^c^		1.55	27.17	^13^C_12_-PCB111/111
		PCB95	323.8834^a^		325.8804^b^, 327.8775^c^		1.55	27.20	^13^C_12_-PCB95/95
		PCB66	293.9165^c^		289.9224^a^, 291.9194^b^		0.77	27.27	^13^C_12_-PCB70/70
		^13^C_12_-PCB155^*^	-		371.8817^b^, 373.8788^c^		1.24	28.09	^13^C_12_-PCB79/162
		PCB155	363.8356^d^		359.8415^b^, 361.8385^c^		1.24	28.11	^13^C_12_-PCB155/155
		PCB60	293.9165^c^		289.9224^a^, 291.9194^b^		0.77	28.51	^13^C_12_-PCB70/70
		PCB84	323.8834^a^		325.8804^b^, 327.8775^c^		1.55	28.70	^13^C_12_-PCB101/101
		PCB90	323.8834^a^		325.8804^b^, 327.8775^c^		1.55	28.83	^13^C_12_-PCB101/101
		^13^C_12_-PCB101^*^	-		337.9207^b^, 339.9178^c^		1.55	28.90	^13^C_12_-PCB111/111
		PCB101	323.8834^a^		325.8804^b^, 327.8775^c^		1.55	28.91	^13^C_12_-PCB101/101
		PCB99	323.8834^a^		325.8804^b^, 327.8775^c^		1.55	29.25	^13^C_12_-PCB95/95
		^13^C_12_-PCB79^**^	-		301.9626^a^, 303.9597^b^		0.77	29.51	-
Scan window/ min	Compound			Qualitative ion		Quantitative ions	Theoretical ratio	*t*_R_/min	Retention time/ quantitation references
	PCB119			323.8834^a^		325.8804^b^, 327.8775^c^	1.55	29.65	^13^C_12_-PCB101/101
	PCB97			323.8834^a^		325.8804^b^, 327.8775^c^	1.55	30.36	^13^C_12_-PCB95/95
	^13^C_12_-PCB111^**^			-		337.9207^b^, 339.9178^c^	1.55	30.67	-
	PCB87			323.8834^a^		325.8804^b^, 327.8775^c^	1.55	30.83	^13^C_12_-PCB123/123
	^13^C_12_-PCB81^*^			-		301.9626^a^, 303.9597^b^	0.77	30.94	^13^C_12_-PCB79/79
	PCB81			293.9165^c^		289.9224^a^, 291.9194^b^	0.77	30.96	^13^C_12_-PCB81/81
	PCB85			323.8834^a^		325.8804^b^, 327.8775^c^	1.55	31.10	^13^C_12_-PCB123/123
	PCB110			323.8834^a^		325.8804^b^, 327.8775^c^	1.55	31.47	^13^C_12_-PCB123/123
	^13^C_12_-PCB77^*^			-		301.9626^a^, 303.9597^b^	0.77	31.68	^13^C_12_-PCB79/79
	PCB77			293.9165^c^		289.9224^a^, 291.9194^b^	0.77	31.70	^13^C_12_-PCB77/77
	PCB151			363.8356^d^		359.8415^b^, 361.8385^c^	1.24	32.26	^13^C_12_-PCB114/155
	PCB135			363.8356^d^		359.8415^b^, 361.8385^c^	1.24	32.53	^13^C_12_-PCB155/155
	PCB149			363.8356^d^		359.8415^b^, 361.8385^c^	1.24	33.13	^13^C_12_-PCB114/155
	^13^C_12_-PCB123^*^			-		337.9207^b^, 339.9178^c^	1.55	33.16	^13^C_12_-PCB111/111
	PCB123			323.8834^a^		325.8804^b^, 327.8775^c^	1.55	33.18	^13^C_12_-PCB123/123
	^13^C_12_-PCB118^*^			-		337.9207^b^, 339.9178^c^	1.55	33.44	^13^C_12_-PCB111/111
	PCB118			323.8834^a^		325.8804^b^, 327.8775^c^	1.55	33.47	^13^C_12_-PCB118/118
	PFK			330.9792		-	-	-	-
33.85-39.40	^13^C_12_-PCB114^*^			-		337.9207^b^, 339.9178^c^	1.55	34.18	^13^C_12_-PCB111/111
	PCB114			323.8834^a^		325.8804^b^, 327.8775^c^	1.55	34.20	^13^C_12_-PCB114/114
	^13^C_12_-PCB188^*^			-		405.8428^b^, 407.8398^c^	1.05	34.28	^13^C_12_-PCB162/162
	PCB188			397.7966^d^		393.8025^b^, 395.7995^c^	1.05	34.32	^13^C_12_-PCB188/188
	^13^C_12_-PCB153^*^			-		371.8817^b^, 373.8788^c^	1.24	34.97	^13^C_12_-PCB162/162
	PCB153/168			363.8356^d^		359.8415^b^, 361.8385^c^	1.24	35.00	^13^C_12_-PCB153/153
	^13^C_12_-PCB105^*^			-		337.9207^b^, 339.9178^c^	1.55	35.28	^13^C_12_-PCB111/111
	PCB105			323.8834^a^		325.8804^b^, 327.8775^c^	1.55	35.31	^13^C_12_-PCB105/105
	PCB141			363.8356^d^		359.8415^b^, 361.8385^c^	1.24	35.91	^13^C_12_-PCB138/138
	PCB137			363.8356^d^		359.8415^b^, 361.8385^c^	1.24	36.32	^13^C_12_-PCB153/153
	^13^C_12_-PCB138^*^			-		371.8817^b^, 373.8788^c^	1.24	36.90	^13^C_12_-PCB111/111
	PCB138			363.8356^d^		359.8415^b^, 361.8385^c^	1.24	36.93	^13^C_12_-PCB138/138
	PCB158			363.8356^d^		359.8415^b^, 361.8385^c^	1.24	37.02	^13^C_12_-PCB138/138
	PCB178			397.7966^d^		393.8025^b^, 395.7995^c^	1.05	37.33	^13^C_12_-PCB167/188
	PCB129			363.8356^d^		359.8415^b^, 361.8385^c^	1.24	37.41	^13^C_12_-PCB138/138
	^13^C_12_-PCB126^*^			-		337.9207^b^, 339.9178^c^	1.55	37.69	^13^C_12_-PCB111/111
	PCB126			323.8834^a^		325.8804^b^, 327.8775^c^	1.55	37.72	^13^C_12_-PCB126/126
	PCB187			397.7966^d^		393.8025^b^, 395.7995^c^	1.05	37.96	^13^C_12_-PCB167/188
	PCB183			397.7966^d^		393.8025^b^, 395.7995^c^	1.05	38.37	^13^C_12_-PCB167/188
	^13^C_12_-PCB162^**^			-		371.8817^b^, 373.8788^c^	1.24	38.56	-
	PCB128			363.8356^d^		359.8415^b^, 361.8385^c^	1.24	38.83	^13^C_12_-PCB167/167
	^13^C_12_-PCB167^*^			-		371.8817^b^, 373.8788^c^	1.24	38.99	^13^C_12_-PCB162/162
	PCB167			363.8356^d^		359.8415^b^, 361.8385^c^	1.24	39.02	^13^C_12_-PCB167/167
	PFK			354.9792		-	-	-	-
39.40-46.10	PCB174			397.7966^d^		393.8025^b^, 395.7995^c^	1.05	39.67	^13^C_12_-PCB180/180
	PCB177			397.7966^d^		393.8025^b^, 395.7995^c^	1.05	40.02	^13^C_12_-PCB180/180
	^13^C_12_-PCB202^*^			-		439.8038^b^, 441.8008^c^	0.89	40.15	^13^C_12_-PCB194/194
	PCB202			431.7576^d^		427.7635^b^, 429.7606^c^	0.89	40.18	^13^C_12_-PCB202/202
	PCB171			397.7966^d^		393.8025^b^, 395.7995^c^	1.05	40.37	^13^C_12_-PCB180/180
	^13^C_12_-PCB156^*^			-		371.8817^b^, 373.8788^c^	1.24	40.57	^13^C_12_-PCB162/162
	PCB156			363.8356^d^		359.8415^b^, 361.8385^c^	1.24	40.60	^13^C_12_-PCB156/156
	PCB201			431.7576^d^		427.7635^b^, 429.7606^c^	0.89	40.76	^13^C_12_-PCB202/202
	^13^C_12_-PCB157^*^			-		371.8817^b^, 373.8788^c^	1.24	40.91	^13^C_12_-PCB162/162
Scan window/ min	Compound			Qualitative ion		Quantitative ions	Theoretical ratio	*t*_R_/min	Retention time/ quantitation references
	PCB157			363.8356^d^		359.8415^b^, 361.8385^c^	1.24	40.93	^13^C_12_-PCB157/157
	^13^C_12_-PCB180^*^			-		405.8428^b^, 407.8398^b^	1.05	41.69	^13^C_12_-PCB162/162
	PCB180/193			397.7966^d^		393.8025^b^, 395.7995^c^	1.05	41.72	^13^C_12_-PCB180/180
	PCB191			397.7966^d^		393.8025^b^, 395.7995^c^	1.05	42.03	^13^C_12_-PCB180/180
	PCB200			431.7576^d^		427.7635^b^, 429.7606^c^	0.89	42.49	^13^C_12_-PCB202/202
	^13^C_12_-PCB169^*^			-		371.8817^b^, 373.8788^c^	1.24	43.25	^13^C_12_-PCB162/162
	PCB169			363.8356^d^		359.8415^b^, 361.8385^c^	1.24	43.27	^13^C_12_-PCB169/169
	^13^C_12_-PCB170^*^			-		405.8428^b^, 407.8398^b^	1.05	43.66	^13^C_12_-PCB162/162
	PCB170			397.7966^d^		393.8025^b^, 395.7995^c^	1.05	43.67	^13^C_12_-PCB170/170
	PCB199			431.7576^d^		427.7635^b^, 429.7606^c^	0.89	44.16	^13^C_12_-PCB202/202
	PCB203			431.7576^d^		427.7635^b^, 429.7606^c^	0.89	44.58	^13^C_12_-PCB202/202
	^13^C_12_-PCB189^*^			-		405.8428^b^, 407.8398^b^	1.05	45.74	^13^C_12_-PCB162/162
	PCB189			397.7966^d^		393.8025^b^, 395.7995^c^	1.05	45.76	^13^C_12_-PCB189/189
	PFK			380.9760		-	-	-	-
46.10-60.00	^13^C_12_-PCB208^*^			-		473.7648^b^, 475.7619^c^	0.77	46.46	^13^C_12_-PCB206/206
	PCB208			465.7187^d^		461.7246^b^, 463.7216^c^	0.77	46.47	^13^C_12_-PCB208/208
	PCB207			465.7187^d^		461.7246^b^, 463.7216^c^	0.77	47.09	^13^C_12_-PCB208/208
	^13^C_12_-PCB194^**^			-		439.8038^b^, 441.8008^c^	0.89	48.07	-
	PCB194			431.7576^d^		427.7635^b^, 429.7606^c^	0.89	48.08	^13^C_12_-PCB194/205
	^13^C_12_-PCB205^*^			-		439.8038^b^, 441.8008^c^	0.89	48.32	^13^C_12_-PCB194/194
	PCB205			431.7576^d^		427.7635^b^, 429.7606^c^	0.89	48.34	^13^C_12_-PCB205/205
	^13^C_12_-PCB206^**^			-		473.7648^b^, 475.7619^c^	0.77	50.44	-
	PCB206			465.7187^d^		461.7246^b^, 463.7216^c^	0.77	50.47	^13^C_12_-PCB206/208
	^13^C_12_-PCB209^*^			511.7199^d^		507.7258^b^, 509.7229^c^	1.16	52.44	^13^C_12_-PCB206/206
	PCB209			499.6797^d^		495.6856^b^, 497.6826^c^	1.16	52.48	^13^C_12_-PCB209/209
	PFK			454.9728		-	-	-	-

*and** indicate extraction internal standard and injection internal standard, respectively. a, b, c and d represent the corresponding characteristic ion fragment types are M, M+2, M+4 and M+6, respectively. PFK (perfluorokerosene) means the lock mass in each function. -: no data.

### 1.2 样品前处理

#### 1.2.1 提取

取市售鱼和赤贝食用部分用高速组织匀浆机捣碎,均质后密封于具塞玻璃瓶中,-18 ℃保存于冰箱。提取前解冻至室温。

准确称取5.00 g样品于小烧杯中,并加入20 g无水硫酸钠搅拌均匀后,转入底部铺有滤膜的34 mL提取池中,然后用约5 g无水硫酸钠分3次清洗小烧杯,一并转入提取池中。加入10 μL 100 ng/mL提取内标PCB-LCS-H(绝对含量为1 ng),用正己烷-二氯甲烷(1∶1, v/v)加速溶剂提取。提取条件为:静提取时间8 min,压力10.3 MPa,提取温度100 ℃,加热时间5 min,循环3次,吹扫时间120 s,淋洗体积60%。

#### 1.2.2 净化

将提取液旋转蒸发至1 mL左右,过酸性硅胶层析柱。层析柱内径为15 mm,自下而上填充1 cm无水硫酸钠、8 g 44%酸性硅胶、1 cm无水硫酸钠,边填充边轻轻敲打,使填料填充均匀密实。使用前用70 mL正己烷预淋洗,活化。将样品完全转移至活化后的层析柱上净化,用90 mL正己烷洗脱。洗脱液旋转蒸发浓缩至1~2 mL后,氮吹浓缩,溶剂转换成壬烷,定容20 μL,加入1 ng进样内标PCB-ISS-H,待上机分析。

### 1.3 色谱-质谱条件

毛细管色谱柱:DB-5MS(60 m×0.25 mm×0.25 μm);进样口温度:270 ℃;传输线温度为280 ℃;柱流速:1.0 mL/min,恒流模式;进样方式:不分流进样;升温程序:初始温度120 ℃,保持1 min,以30 ℃/min的升温速率升至180 ℃,以2 ℃/min升至210 ℃,保持1 min,再以2.5 ℃/min升至310 ℃,保持1 min;载气:氦气,纯度≥99.9999%;进样体积:1.0 μL。

双聚焦磁质谱,静态分辨率为10000,动态分辨率>8000(分辨率采用单峰5%峰谷定义),并至少可稳定12 h以上。离子源温度:280 ℃;电子能量:35 eV;捕集电流:650 μA;加速电压:7950 V;光电倍增管电压:350 V^[13,14]^;选择离子监测(SIM)模式。各PCBs在HRGC-HRMS上的窗口划分、特征离子、定量特征离子理论丰度比、保留时间、保留时间参考和定量参考见表1。

### 1.4 定性定量方法

在给定色谱-质谱条件下,获得样品色谱图和质谱图,根据保留时间和特征离子丰度比进行定性,以平均相对响应因子法(RRF)进行定量。由校准溶液测定化合物的RRF,并计算平均值。其中,35种PCBs使用各自^13^C标记的提取内标定量,其余采用保留时间接近的^13^C标记的提取内标定量;除了一氯联苯、七氯联苯和十氯联苯提取内标分别采用二氯联苯(^13^C-PCB9)、六氯联苯(^13^C-PCB162)和九氯联苯(^13^C-PCB206)进样内标定量,其余提取内标均用相同氯代水平的进样内标定量。化合物信息及其氯代水平详细见表1和附表1。

## 2 结果与讨论

### 2.1 色谱分离和质谱条件的选择

PCBs的同类物属于非极性物质,因此本实验选择非极性固定液的石英毛细管柱DB-5MS(60 m×0.25 mm×0.25 μm),考察了不同升温程序对82种目标物分离情况的影响。发现采用1.3节所述的升温程序时,色谱分离效果良好,如图1所示的八氯联苯PCB194和PCB205,两者可实现基线分离。82种PCBs标准溶液(200 ng/mL)总离子流色谱图见图2。其中PCB4与PCB10、PCB28与PCB31、PCB153与PCB168、PCB180与PCB193在DB-5MS色谱柱的分配系数相同,且质谱碎片离子一致,无法实现分离并定性,因此被视为共流出,定量结果为两者的总和。

**图1:**
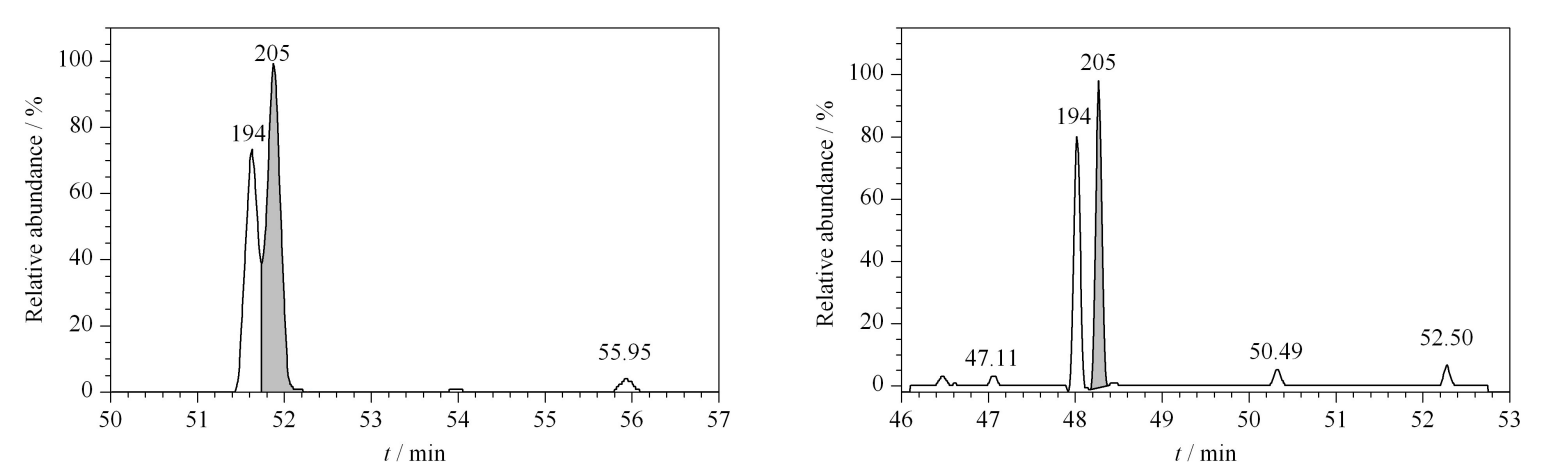
PCB194和PCB205的分离

**图2:**
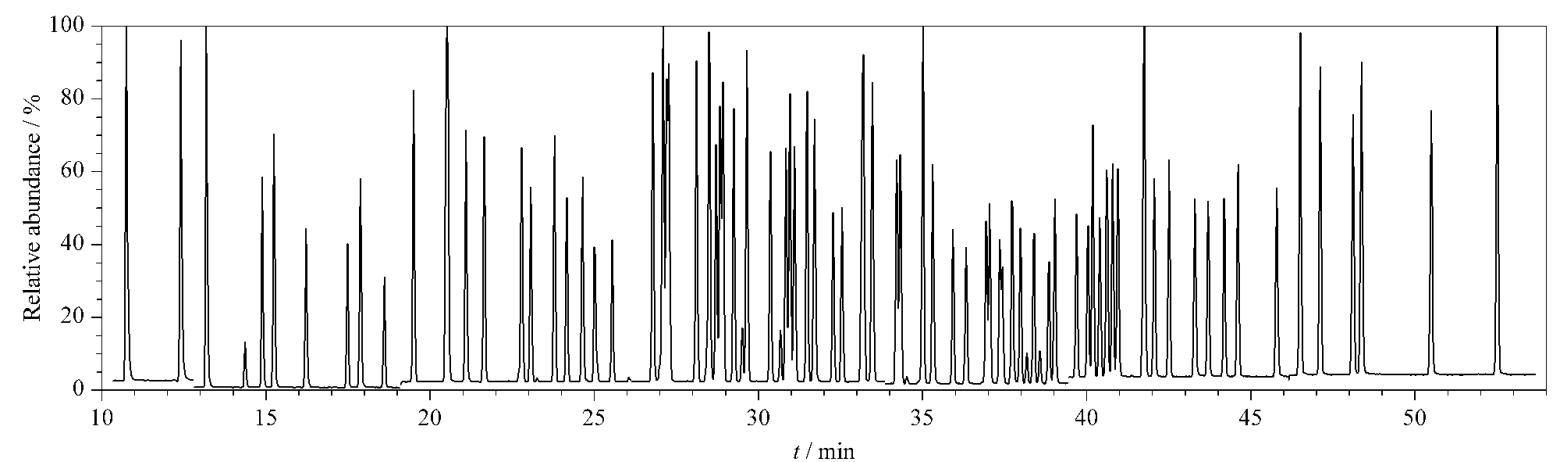
PCBs标准溶液(200 ng/mL)的总离子流色谱图

根据优化好的升温程序条件,参考文献[14],对PCBs的特征碎片离子进行窗口划分,总共划分了7个监测窗口(见表1)。第一个窗口监测一氯联苯,第二个窗口监测二氯联苯和三氯联苯,第三个窗口监测部分三氯联苯、四氯联苯和五氯联苯,第四个窗口监测四氯联苯、五氯联苯和六氯联苯,第五个窗口监测五氯联苯、六氯联苯和七氯联苯,第六个窗口监测六氯联苯、七氯联苯和八氯联苯,第七个窗口监测八氯联苯、九氯联苯和十氯联苯。选择的质谱质量轴参考物为全氟代煤油(PFK),每个窗口PFK锁定离子接近或在该窗口分析物碎片离子范围内,且峰响应较强^[13]^。

### 2.2 实验条件

#### 2.2.1 洗脱体积的确认

参照EPA 1688A方法,采用8 g酸性硅胶层析柱净化样品。由于同位素标记的化合物的物化性质与其天然化合物相同,因此在确认洗脱体积时,直接使用相应的^13^C标记化合物^[14]^。酸性硅胶层析柱填充时需紧实,可有效提高净化效果。层析柱经70 mL正己烷预淋洗后,加入1 ng提取内标,然后依次用10、20、20、20、20 mL正己烷洗脱,并分别收集各流分,共5个流分。发现化合物大部分集中在流分1和2中,而流分5中化合物回收率为0~0.4%,因此选择正己烷的洗脱体积为90 mL。

#### 2.2.2 样品提取方式的确定

EPA 1688A方法推荐使用索式提取装置提取样品18~24 h^[14]^。索氏抽提法是传统固体样品中微量、痕量有机污染物的提取方法,至今仍被广泛使用,在分析PCBs方面的报道也有很多,如沉积物、土壤和动植物组织等^[15,16]^。但是索氏抽提法溶剂消耗量大且耗时长,鉴于此,我们考虑采用水平振荡法和加速溶剂提取法提取样品。

各取3份贝类样品,加入1 ng提取内标后,分别用水平振荡法和加速溶剂提取法进行提取。水平振荡法提取条件:30 mL正己烷-二氯甲烷(1∶1, v/v)振荡提取2次,每次40 min,频次200次/min。加速溶剂提取法条件为1.2.1节所述。经过实验发现,振荡提取的回收率虽然在可接受范围内,但由于振荡加速溶剂挥发使瓶内压力增加,瓶塞易松动或冲开溅出溶剂,操作难度较大,导致回收率不易控制,且超过一半提取内标回收率的RSD≥30%,重复性差。加速溶剂提取法实现了样品全自动加压加热溶剂提取,不仅解放人力,节约试剂,而且易于操作,回收率易于控制且重复性好,提取内标回收率为44.5%~135%, RSD为1.3%~19%(见附表2),达到EPA 1688A方法回收率(^13^C-PCB1、^13^C-PCB3回收率为15%~150%,其余物质为25%~150%)和RSD≤50%的要求。因此,最终选择加速溶剂提取法进行样品提取。

#### 2.2.3 提取溶剂的选择

实验在文献^[9,17,18]^基础上,确定ASE的提取条件为:静提取时间8 min,压力10.3 MPa,提取温度100 ℃,加热时间5 min,循环3次,吹扫时间120 s,淋洗体积60%。在此条件下,对提取溶剂进行了选择。

多氯联苯为弱极性物质,根据相似相溶原理,多采用二氯甲烷、正己烷、丙酮或其混合溶剂为提取溶剂。按1.2.1 ASE提取条件和1.2.2净化条件,研究了正己烷-丙酮(1∶1, v/v)和正己烷-二氯甲烷(1∶1, v/v)对PCBs的提取效率。结果发现两者对PCBs的提取效率分别为35.9%~136%和33.1%~128%,均满足EPA 1688A的要求。考虑到水产品中含有大量油脂,干扰组分复杂,丙酮是强极性溶剂,提取目标物的同时也会提取更多杂质,加重样品的净化负担。因此,选择正己烷-二氯甲烷(1∶1, v/v)为提取溶剂。

### 2.3 平均相对响应因子、相关系数和方法检出限

将PCBs系列标准溶液(0.1、0.5、2.0、10.0、40.0和200 ng/mL)进样,采用同位素稀释法计算目标物和提取内标的RRF及其RSD。结果表明,线性相关系数(*r*^2^)均>0.99(见附表3), RFF的RSD≤20%,表明各PCBs的RRF结果稳定,可进行定量计算。按照HJ 168-2020要求,计算方法检出限(MDL),按照样品分析的全流程,以鱼/贝类的实际样品为基质空白,重复测定7次,按公式MDL=*t*_(_*_n_*_-1, 0.99)_×*S*计算方法检出限(*n*=7时,*t*_(_*_n_*_-1, 0.99)_=3.143; *S*为标准偏差),分别得到鱼类和贝类的MDL。未检出的物质采用仪器软件以3倍信噪比计算检出限。最终确定生物样品的方法检出限为0.02~3 pg/g,结果见附表3。

### 2.4 方法回收率和精密度

在优化后的实验条件下,分别取鱼肉和赤贝作加标回收试验,加入低(0.4 ng)、高(3.6 ng)水平的天然化合物PCB-PAR-H,每个水平重复测定7次,验证方法的回收率和精密度。从附表4可见,低水平加标时,鱼和贝类平均回收率分别为71.3%~139%和76.9%~143%, RSD分别为2.1%~14%和4.5%~14%;高水平加标时,鱼和贝类平均回收率分别为77.6%~141%和82.2%~131%, RSD分别为1.4%~9.4%和1.7%~11%。表明方法的准确度和精密度均能达到满意结果。

### 2.5 实际样品测定

采用本文方法对北京市某农贸市场市售新鲜鱼、新鲜赤贝各1种进行了分析测定。新鲜鱼中PCBs单体含量范围为未检出~13.8 pg/g, 12种类二噁英类PCBs含量为12.6~17.5 pg/g, 6种指示性PCBs含量为30.9~46.7 pg/g, 82种PCBs总含量为174~251 pg/g,回收率为42.1%~121%;新鲜赤贝中PCBs单体含量范围为未检出~54.1 pg/g, 12种类二噁英类PCBs含量为30.4~74.5 pg/g, 6种指示性PCBs含量为51.8~62.1 pg/g, 82种PCBs总含量为282~672 pg/g,提取回收率为38.2%~113%。

## 3 结论

本文利用加速溶剂提取结合酸性硅胶柱净化的样品前处理技术和同位素稀释法定量方式,采用HRGC-HRMS进行测定,建立了生物样品中82种多氯联苯的检测方法。该方法使用ASE进行样品提取,操作简单;采用酸性硅胶柱净化,去除生物样品中脂肪的同时对样品进行净化,有效去除干扰物;利用同位素稀释法定量,保证了方法的准确性。该方法前处理简单,回收率好,灵敏度高,重复性佳,可为国内外更加全面具体分析鱼和贝类等水产品受PCBs污染情况和开展生物监测提供有效的技术支持。同时,该方法也可以更好地服务于相关生态环境管理及履行《斯德哥尔摩公约》。
